# Two-Photon Microscopy Allows Imaging and Characterization of Cochlear Microvasculature In Vivo

**DOI:** 10.1155/2015/154272

**Published:** 2015-03-30

**Authors:** Friedrich Ihler, Mattis Bertlich, Bernhard Weiss, Steffen Dietzel, Martin Canis

**Affiliations:** ^1^Department of Otorhinolaryngology, University of Göttingen Medical Center, Georg-August-Universität Göttingen, Robert-Koch-Strasse 40, 37099 Göttingen, Germany; ^2^Walter-Brendel-Zentrum für Experimentelle Medizin (WBex), Ludwig-Maximilians-Universität München, Marchioninistrasse 15, 81377 München, Germany

## Abstract

Impairment of cochlear blood flow has been discussed as factor in the pathophysiology of various inner ear disorders. However, the microscopic study of cochlear microcirculation is limited due to small scale and anatomical constraints. Here, two-photon fluorescence microscopy is applied to visualize cochlear microvessels. Guinea pigs were injected with Fluorescein isothiocyanate- or Texas red-dextrane as plasma marker. Intravital microscopy was performed in four animals and explanted cochleae from four animals were studied. The vascular architecture of the cochlea was visualized up to a depth of 90.0 ± 22.7 *μ*m. Imaging yielded a mean contrast-to-noise ratio (CNR) of 3.3 ± 1.7. Mean diameter in vivo was 16.5 ± 6.0 *μ*m for arterioles and 8.0 ± 2.4 *μ*m for capillaries. In explanted cochleae, the diameter of radiating arterioles and capillaries was measured with 12.2 ± 1.6 *μ*m and 6.6 ± 1.0 *μ*m, respectively. The difference between capillaries and arterioles was statistically significant in both experimental setups (*P* < 0.001 and *P* = 0.022, two-way ANOVA). Measured vessel diameters in vivo and ex vivo were in agreement with published data. We conclude that two-photon fluorescence microscopy allows the investigation of cochlear microvessels and is potentially a valuable tool for inner ear research.

## 1. Introduction

The pathogenesis of most inner ear disorders is still largely unknown and subject to controversial discussion [1]. Hearing depends on a homeostasis of fluid and ions in the inner ear which is maintained by cochlear microcirculation [2]. Inner ear blood supply is provided by the labyrinthine artery as a branch of the anterior inferior cerebellar artery. Its terminal vessel, the spiral modiolar artery, radiates arterioles to the lateral cochlear wall forming a capillary system in the stria vascularis which lies within the spiral ligament [3]. Impairment of cochlear blood flow has been considered as a factor in the pathophysiology of various kinds of inner ear disorders [4–6] like noise-induced hearing loss and presbycusis [7]. Owing to the typical clinical features, this is also discussed as the cause for sudden sensorineural hearing loss [7, 8].

However, in humans direct evidence for this hypothesis is lacking due to limits for clinical investigation. Difficulties result from the complexity and the hidden localization of the cochlea within the temporal bone, thus precluding visualization of cochlear microcirculation. Well-established clinical imaging modalities such as magnetic resonance imaging (MRI) [9], single photon emission computed tomography (SPECT) [10], and ultrasound imaging [6] have been adopted for vascular imaging but lack either sufficient spatial resolution, satisfactory contrast, or both to be effective for cochlear microvascular imaging.

Data about the configuration of cochlear blood supply had been gathered at first by ex vivo examination of cast or dissected preparations of animal and human cochleae [3, 11–13]. Intravital microscopy was developed to study cochlear microvessels in guinea pigs in vivo [14, 15], and various animal models were established to assess cochlear blood flow. Methods applied include laser-Doppler flowmetry [16, 17], microelectrode oxygen tension determination [18], labeled [19] or unlabeled [20] microsphere techniques, laser speckle contrast imaging in combination with Doppler optical microangiography [21], and intravital fluorescence microscopy [14, 22–24]. Conventional fluorescence microscopy provides the best spatial resolution of these techniques and thereby allows selective quantification of blood flow in stria vascularis capillaries. However, it requires ablation of the overlying bone to access cochlear microvessels. Moreover, a specific investigation is limited to capillaries, which lie in the outermost part of the cochlear turn in the spiral ligament. Cochlear radiating arterioles cannot be investigated directly so far. This is because of their anatomical location deep within the ceiling of the scala vestibuli [3, 25]. Moreover, conventional fluorescence microscopy requires preparatory thinning of the cochlear lateral wall [14], with so far undefined but potentially severe consequences on local physiology [26].

Two-photon laser scanning fluorescence microscopy was first described by Denk and coworkers [27]. It is a light microscopy technique that allows in vivo imaging up to a depth of one millimeter from the surface of a specimen in some tissues [28–30], providing subcellular resolution and good light penetration as well as low phototoxicity [31, 32]. An important constraint of conventional light microscopy is scattering and absorption of photons. Multiphoton fluorescence microscopy overcomes this by exciting with near-infrared light in frequencies that have superior scattering characteristics. Beyond this, absorption of biological molecules is minimal at the frequencies applied, allowing deeper tissue penetration [33]. The application of two simultaneous photons with high frequency and low energy additionally allows a more focused approach with imaging confined to small volumes [34]. Thus, two-photon microscopy may circumvent invasive preparation while enabling volumetric visualization of a specimen.

In the present study we applied two-photon microscopy to the cochlea of guinea pigs to test whether it allows visualization of the cochlear microvasculature without prior removal of the overlaying cochlear bone.

## 2. Material and Methods

### 2.1. Experimental Setup

Six female adult albino Dunkin-Hartley guinea pigs (weight range, 250–300 g) were obtained from Charles River Laboratories (Sulzfeld, Germany). The experiments were performed according to Bavarian state regulations for animal experimentation and were approved on July 27, 2006, by the District Government of Upper Bavaria (animal license number: 55.2-1-54-2531-57-06).

Guinea pigs were anesthetized using intraperitoneal injections of ketamine 85 mg/kg b.w. (Ketavet Parke-Davis, Berlin, Germany) and xylazine 8.5 mg/kg b.w. (Rompun; Bayer, Leverkusen, Germany). A surgical level of anaesthesia was maintained by supplementary half doses of ketamine and xylazine injected every 45 min. This protocol was shown earlier to reliably maintain systemic blood pressure [22].

During the experiments, replacement fluid was infused (NaCl, 8 *μ*L/100 g b.w./min) to maintain renal blood flow and to compensate for fluid loss encountered during anaesthesia. The animals were placed on a thermostatically controlled heating pad which maintained temperature at 38°C. Heart rate and oxygen saturation were continuously monitored by pulsoxymetry. During surgical procedures, middle arterial pressure was 67 ± 12 mmHg and heart rate was 230 ± 26/min on average (*n* = 6). A polyethylene catheter (PE 50) was placed in the right jugular vein for intravenous injections. Head fixation of the animals was realized by a custom-made head holder as described previously [22].

### 2.2. Surgical Preparations

**Figure 1:**
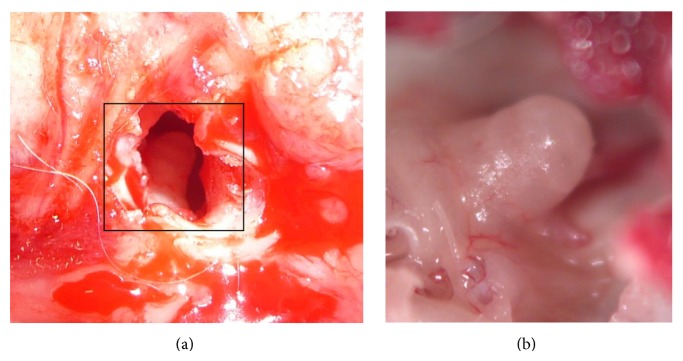
Exposure of a guinea pig cochlea. (a) The right bulla was opened through a lateral and ventral approach to allow access to the cochlea (box) for two-photon microscopy. (b) Explanted cochlea in a larger magnification.

Through a postauricular incision, the right auditory bulla was opened by a dorsolateral approach. The auricle, overlying soft tissue, the lateral bony part of the bulla including the tympanic membrane, posterior annulus, and tensor tympani tendon as well as parts of the bony bulla were removed. To provide access of the objective to the cochlea, the perpendicular portion of the ramus of the mandibular was sacrificed as well as the middle ear ossicles in order to allow a direct line of visualization. Mucosa and mucosal vessels overlying the second and the third turns of the cochlea were gently wiped off by a piece of gel foam. Anatomic landmarks such as the facial nerve and the semicircular canals were identified (Figure 1).

Following in vivo measurements, animals were euthanized by intraperitoneal injection of 800 mg/kg b.w. of sodium pentobarbital. For the examination of explanted cochleae, after systemic injection of fluorescent dye and euthanasia, the petrous part of the temporal bone was removed as a whole. Then, the cochlea was exposed by careful dissection of the bulla. The specimen was studied unfixed in a solution of 0.9% NaCl.

### 2.3. Two-Photon Microscopy

Fluorescein isothiocyanate- (FITC-) labeled dextran (order number 46947; molecular weight 500 kDa; 0.05 to 0.1 mL of a 5% solution in 0.9% NaCl; Sigma, Deisenhofen, Germany) or Texas red-labeled dextran (order number D1830; molecular weight 70 kDa; 1.0 mL of a 5% solution in 0.9% NaCl; Life Technologies, Carlsbad, CA, USA) was injected intravenously as a plasma marker to visualize cochlear microcirculation. Multiphoton microscopy was performed on a TriMScope (LaVision BioTec, Bielefeld, Germany) described elsewhere [35, 36].

Two water immersion objectives were used for image acquisition, either 20x (numerical aperture 0.95, working distance 2 mm, field number 22 mm, and field of view in current study 0.5 mm × 0.5 mm) or 10x magnification (numerical aperture 0.3, working distance 3.5 mm, field number 26.5 mm, and field of view in current study 1 mm × 1 mm). 0.9% NaCl or ultrasound gel was applied as immersion liquid. Excitation was achieved with 800, 860, or 1180 nm.

### 2.4. Image Processing and Analysis

Two-dimensional images of fluorescence intensity (*XY*) in successive depths were processed as three-dimensional stacks (*XYZ*) in Tagged Image File Format (TIFF) by ImageJ 1.47v [37], the distribution Fiji [38], or Imaris 7.6 (Bitplane, Zürich, Switzerland). Stitching of neighboring stacks to one was performed with a plug-in for Fiji [39].

Vessels were classified by their branching characteristics as radiating arteriole before the first offshoot or as capillary thereafter. Radial arterioles have been described to run within the spiral osseous lamina perpendicular to the midmodiolar axis of the cochlea while capillaries are branching from them in the lateral cochlear wall with an angle of about 60 degrees [3]. These characteristics were readily identifiable in our experimental setup.

Depth of visualization was determined by identifying the deepest visible vessel in each stack and averaging the depth value across stacks. Vessel borders could be easily identified and blurred edges constituted no major obstacle due to viable resolution. However, to rule out any remaining variability, values for vessel diameters were obtained by averaging 5 independent measurements along the visible sector on each identified vessel, thereby correcting for variations along the vessel axis. The mean coefficient of variation of the individual measurements of diameter for the respective vessels was 0.05 (standard deviation 0.04, range 0.01–0.19), signifying a low variation.

Contrast-to-noise ratio (CNR) was calculated by measuring mean grey value (M) and standard deviation of grey value (SD) in representative regions of interest within (M_*W*_, SD_*W*_) and outside (M_*O*_, SD_*O*_) the studied vessels by application of the formula

(1) $$\begin{array}{c} \text{CNR}=\frac{\left({\text{M}}_{W}-{\text{M}}_{O}\right)}{\text{sqrt}\left({{\text{SD}}_{W}}^{2}+{{\text{SD}}_{O}}^{2}\right)} \end{array}$$

as described before [40, 41].

### 2.5. Statistical Analysis

Statistical analysis was performed using SigmaPlot 12 (Systat Software, Chicago, IL, USA). To identify differences greater than expected by chance, result values were compared using two-way analysis of variance (ANOVA). Thereby, vessel diameter and contrast-to-noise ratio (CNR) were analysed for differences dependent on vessel type (capillary or arteriole), specimen (in vivo measurement or explant), and dye (FITC or Texas red-dextran). To correct for multiple comparisons, the Holm-Sidak method was applied. A *P* value < 0.05 was considered to be statistically significant.

## 3. Results

**Figure 2:**
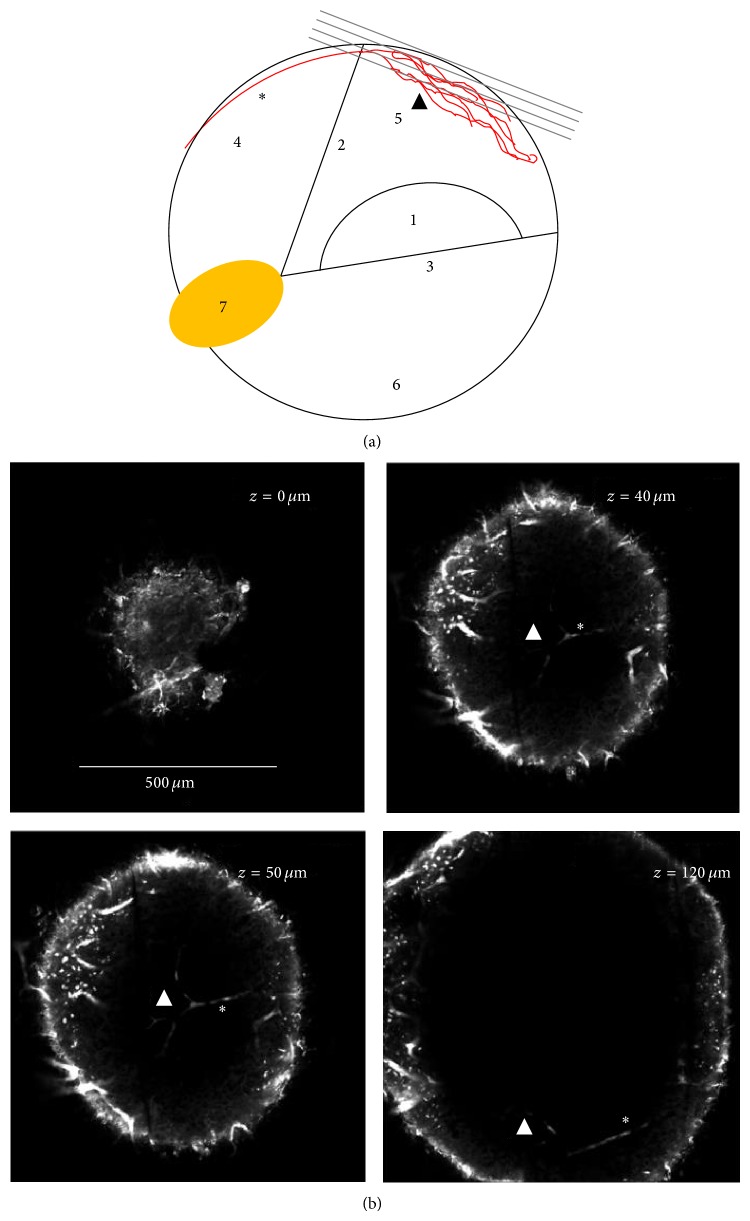
Three-dimensional two-photon imaging of an explanted cochlea. (a) The schematic drawing shows the location of successive *XY*-images of the guinea pig cochlear lateral wall (dark grey) in relation to neighboring structures in a cross section of a cochlear turn. The images are recorded along the *Z*-direction using two-photon microscopy after application of Texas red-labeled dextran and excitation with 1180 nm. (b) Four of the equidistantly acquired optical sections were selected for representation in this figure to show specific features. A strong autofluorescence signal visualizes the bony capsule of the cochlea. ∗, radiating arteriole; ▲, stria vascularis capillaries; 1, organ of Corti; 2, membrane of Reissner; 3, basilar membrane; 4, scala vestibuli; 5, scala media; 6, scala tympani; 7, spiral ganglion.

After surgical exposure of the cochlea (Figure 1), intravital observation of cochlear microcirculation with two-photon laser scanning microscopy was feasible through the intact cochlear bony wall without ablation of the bone. Visualization was possible up to a depth of 90.0 ± 22.7 *μ*m from the outer surface of the bone. Optical sections of the stria vascularis vasculature were recorded along the *Z*-direction from the third turn (Figure 2).

**Figure 3:**
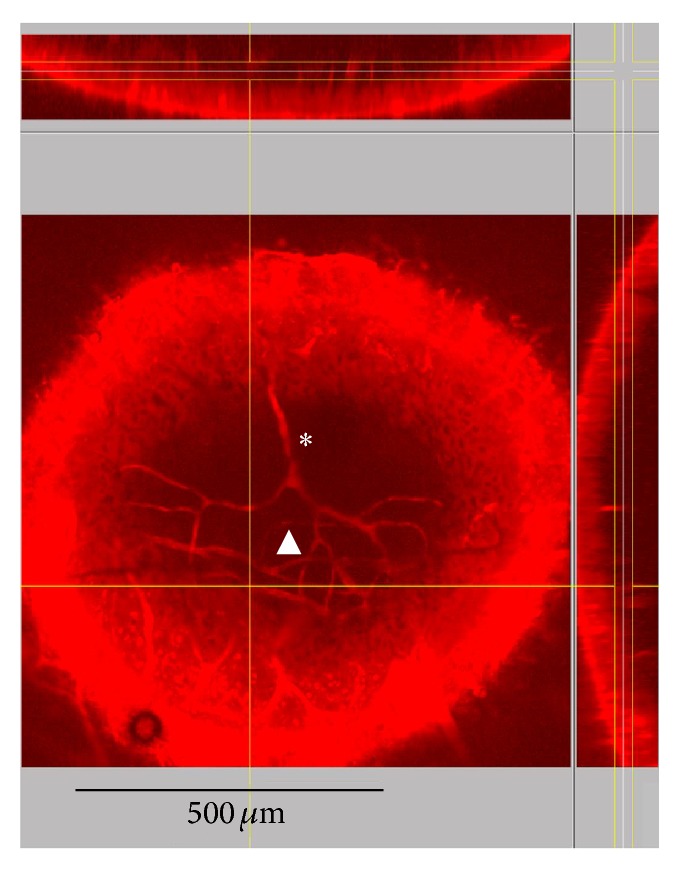
Vascular architecture of the guinea pig cochlea. Visualization of microvessels in an explanted cochlea by two-photon microscopy. The main image shows an *XY*-projection of those sections which are between the vertical yellow lines in the *YZ*-section on the right. This *YZ*-section is placed at the vertical yellow line in the main image. The horizontal yellow line indicates the position of the *XZ*-section shown at the top, mostly covered by the inset. ∗, radiating arteriole; ▲, stria vascularis capillaries.

Four cochleae from four animals were examined by intravital two-photon microscopy. From the respective images, six arterioles and six capillaries were selected for quantification. As an alternative to intravital microscopy, four explanted cochleae from four animals were subjected to two-photon microscopy. The elimination of animal movement allowed recording of higher resolution images with slower scan rates (Figure 3).

Regarding image quality, two-photon microscopy yielded a mean contrast-to-noise ratio (CNR) of 3.3 ± 1.7 in all images. CNR for images obtained from explanted cochleae was 3.7 ± 1.7 while CNR was 3.1 ± 1.8 in in vivo images. Fluorescein isothiocyanate (FITC) as a fluorescent dye resulted in a CNR of 3.6 ± 1.6 while CNR of images obtained with Texas red was 3.1 ± 1.8. Image quality with Texas red was higher in images from explanted cochleae compared to in vivo images (*P* = 0.015). Contrastingly, in measurements on living animals, FITC was superior to Texas red (*P* = 0.022). An overview of contrast-to-noise values is given in Table 1.

Mean diameter in vivo was 16.5 ± 6.0 *μ*m for six arterioles and 8.0 ± 2.4 *μ*m for six capillaries. The difference was statistically significant (*P* < 0.001, two-way ANOVA). An example of a time series of a *XY*-image is presented in Video 1 (see Video 1 in Supplementary Material available online at http://dx.doi.org/10.1155/2015/154272). In freshly explanted, unfixed cochleae, the diameter of five radiating arterioles and five capillaries was measured with 12.2 ± 1.6 *μ*m and 6.6 ± 1.0 *μ*m, respectively. This difference was statistically significant (*P* = 0.022, two-way ANOVA). Although a change in diameter might be expected for vessels in explants, for example, due to postmortal shrinking, the difference to in vivo diameters was not significant in the present data. An overview of the vessel diameters obtained can be found in Table 2.

## 4. Discussion

Conventional intravital fluorescent microscopy after surgical reduction of the bony cochlear lateral wall was to date the only published approach for quantification of vessel count, diameter, length, density, permeability, and blood flow velocity in cochlear microcirculation [14, 22–24]. However, surgical preparation of the cochlear lateral wall as required for conventional microscopy bears the risk of disturbing local physiology [26], thus potentially invalidating the measurements performed. Here we demonstrate that investigations are possible through the intact cochlear lateral wall with two-photon fluorescence microscopy. We were able to visualize the three-dimensional anatomy of the cochlear vasculature, assess vessel diameters in vivo and ex vivo, and show that in principle blood flow measurements are possible with this approach.

In microcirculatory experiments under anesthesia, an independent effect of anesthetics on microvessels is to be discussed. Xylazine and ketamine are known to provide reliably stable cardiorespiratory parameters during surgical experiments [42]. There are currently no studies focusing explicitly on the effect of this combination on cochlear microcirculation. In an experimental setup employing a dorsal skinfold chamber, ketamine was supposed to cause a constrictive state during induction and maintenance of anesthesia compared to the awake state [43]. Contrasting reports have shown a negligible effect of xylazine on vessel diameters [44]. The anesthesia protocol applying ketamine and xylazine in the present report has been shown to provide stable narcosis without confounding experiments in several studies to date [22, 45–50].

The definition of cochlear capillaries is an issue not yet finally resolved. A considerable fraction of the studies on cochlear microvasculature do not elaborate on the classification system applied, most likely since in vivo only capillaries were visible [11–14, 24, 51]. Preparations of the cochlea by cast in earlier studies allowed following the complete vessel tree and thereby identifying different segments. The radiating arterioles have been described as singular vessels branching from the spiral modiolar artery and running perpendicularly from the midmodiolar axis within the spiral lamina towards the cochlear lateral wall [3, 52], where they feed into the stria vascularis in regular intervals [11, 52]. Capillaries have been described to branch off these vessels with an angle of about 60 degrees [3]. Other investigators acknowledged the classification of cochlear microvessels as an issue in in vivo experiments but concluded that visual classification by known branching characteristics is a method with acceptable accuracy [15].

A mean contrast-to-noise ratio (CNR) of 3.3 ± 1.7 in all images of the present study represents an acceptable level of quality. Other optical or combined approaches that attempted to achieve subsurface imaging in vivo reported a CNR of 1.95 for a penetration depth of up to 7 mm [41] or 3.5 to 10 for up to 8 mm [53]. However, no previous study had to deal with the challenging situation of bone as an optically dense substance overlying a liquid as in the guinea pig cochlea in the present study.

Previously published data stated the diameter of stria vascularis capillaries in guinea pigs from 7.5 to 16.0 *μ*m in vivo [15, 24, 51] and 9.2 to 21.8 *μ*m ex vivo [11–13], while radiating arterioles were reported to measure 12.0 to 19.3 *μ*m ex vivo [3, 12, 52]. Our results of 8.0 ± 2.4 *μ*m for capillaries in vivo and 12.2 ± 1.6 *μ*m for arterioles ex vivo are well in line with those earlier reports. The diameter of stria vascularis capillaries in unfixed, freshly explanted cochleae of 6.6 ± 1.0 *μ*m ex vivo in the present study was considerably smaller than values published on fixed material. An explanation for that could be an enlargement of the vasculature by increased pressure that was discussed to occur during perfusion with fixation agents [12]. No prior in vivo data on radiating arterioles are available, most likely due to their location within the roof of the scala vestibuli inaccessible for traditional methods. Therefore, this was assessed with 16.5 ± 6.0 *μ*m in vivo for the first time in the present report.

There is an ongoing debate where blood flow regulation in the cochlea takes place. Arteriolar resistance vessels surrounded by smooth muscle cells have been suggested to play an important part [2]. In contrast, capillaries show no smooth muscle cells but contain a high density of pericytes [2, 54] which exhibit vasocontractility under both in vivo and in vitro conditions [2, 54, 55] and thereby may be an important factor in the regulation of cochlear microcirculation [2, 54]. Conventional intravital microscopy lacks the depth resolution that is crucial for characterizing three-dimensional microvascular morphology of the vascular bed on a cellular level, thereby making two-photon microscopy a valuable novel approach to address those questions. A possible restriction, however, might be imaging speed, as visualization by a laser-scanning application like two-photon microscopy might limit the image section accessible for real-time imaging [33]. A further limitation is the required amount of surgical removal of tissue that does not allow repeated assessments over several days or longer. However, during the experiments the surgical procedures and consecutive measurements were tolerated quite well and cardiovascular parameters were stable.

The feasibility of observation of cochlear vasculature through the intact cochlear bony wall as demonstrated here bears the potential to develop into a valuable animal model for research on inner ear microcirculation. In addition to superior penetration depth compared to conventional confocal microscopy [29], two-photon excitation minimizes photobleaching, the destruction of fluorophores, and tissue damage by phototoxicity, ameliorating further problems of earlier approaches. Two-photon-excitation line-scanning approaches [56–59] may allow measurement of red blood cell velocity, shear stress, and related hemodynamic parameters of cochlear blood flow.

## 5. Conclusion

Two-photon microscopy allows the differentiation of radiating arterioles and stria vascularis capillaries of the cochlea in vivo and ex vivo and has potentially broad applications in imaging and characterization of cochlear microcirculation in health and disease models.

## Supplementary Material

Video 1: Intravital multi-photon recording of the guinea pig cochlear microvasculature. A radiating arteriole (right) is feeding into capillaries of the stria vascularis (left) in the cochlear lateral wall. A sequence of 22 seconds is shown. Scale bar is 40 *μ*m. Gamma value was set to 2, to allow simultaneous visualization of darker and brighter areas.

## Figures and Tables

**Table 1:** Image quality in two-photon microscopy of cochlear microvessels ex vivo and in vivo.

Specimen/dye		FITC	Texas red	*P* (two-way ANOVA)
Ex vivo examination (explanted cochlea)	Number of vessels	5	5	
	CNR	3.0 ± 1.0	4.4 ± 2.1	=0.161

In vivo examination	Number of vessels	6	6	
	CNR	4.2 ± 2.0	2.0 ± 0.4	*=0.022* ^*^

**P** ** (two-way ANOVA)**		=0.227	*=0.015* ^*^	

ANOVA: analysis of variance; CNR: contrast-to-noise ratio; FITC: fluorescein isothiocyanate; ^*^difference statistically significant with *P* < 0.05.

**Table 2:** Diameter of cochlear microvessels ex vivo and in vivo by two-photon microscopy.

Specimen/vessel		Arterioles	Capillaries	*P* (two-way ANOVA)
Ex vivo examination (explanted cochlea)	Number of vessels	5	5	
	Diameter [*µ*m]	12.2 ± 1.6	6.6 ± 1.0	*=0.022* ^*^

In vivo examination	Number of vessels	6	6	
	Diameter [*µ*m]	16.5 ± 6.0	8.0 ± 2.4	*<0.001* ^*^

**P** ** (two-way ANOVA)**		=0.060	=0.512	

ANOVA: analysis of variance; ^*^difference statistically significant with *P* < 0.05.
